# Study of the effect of oral gabapentin used as preemptive analgesia to attenuate post-operative pain in patients undergoing abdominal surgery under general anesthesia

**DOI:** 10.4103/1658-354X.71409

**Published:** 2010

**Authors:** Harshel G. Parikh, Sananta Kumar Dash, Chitra B. Upasani

**Affiliations:** *Department of Anesthesia & Critical Care, Grant Medical College & Sir J.J. Group of Hospitals, Mumbai-08, India*

**Keywords:** *Abdominal surgery*, *gabapentin*, *pre-emptive analgesia*, *post-operative pain*

## Abstract

**Aims:**

To study the effect of oral gabapentin used as preemptive analgesia to attenuate post operative pain in patients undergoing abdominal surgery under general anesthesia.

**Materials and Methods:**

In a randomized double blind study, 60 patients were divided into two groups. Group A received 600mg gabapentin and group B oral received placebo 1 h prior to surgery. Anesthesia was induced with Propofol 2 mg/kg and Vecuronium 0.1mg/kg and maintained with 60% N_2_O in O_2_ and Vecuronium 0.02 mg/kg. All cases were given Fentanyl 2*µ*g/kg as pre medication and a repeat dose 1*µ*g/kg at the end of the first hour. Assessment of post-operative pain was made with the visual analog score (VAS) at extubation (0 h), 2, 4, 6, 12, and 24 h post-operatively. Post-operative analgesia was provided with intravenous Tramadol. The first dose was given in the Post Anesthesia Care Unit as 2mg/kg, and repeated at 8 and 16 h. Rescue analgesia was given with Diclofenac 1.5mg/kg, slow intravenous. The number of doses of rescue analgesia in both the groups was noted.

**Results:**

The VAS scores at 0, 2, 4, 6, 12, and 24 h were 1.9 *vs*. 2.4 (*P*=0.002), 2.3 *vs*. 3.0 (*P*=0.000), 3.2 *vs*. 3.7 (*P*=0.006), 2.9 *vs*. 4.4 (*P*=0.000), 3.6 *vs*. 4.6 (*P*=0.000), and 3.7 *vs*.4.6 (*P*=0.000), respectively. Numbers of patients requiring rescue analgesia with Diclofenac were 3 *vs*. 14 (*P*=0.004).

**Conclusion:**

A single oral dose of gabapentin given pre-operatively enhanced the analgesic effect of Tramadol as it also reduced the requirement of rescue analgesia with Diclofenac.

## INTRODUCTION

Post-operative pain is not purely nociceptive in nature and may consist of inflammatory, neurogenic, and visceral components. Therefore, multimodal analgesic techniques utilizing a number of drugs acting on different analgesic mechanisms are becoming increasingly popular.[1] Post-operative pain affects recovery from surgery and anesthesia. The use of opioids by patient-controlled analgesia is popular, but limited by side-effects and by the fact that certain types of pain respond poorly to opioids.[2] Because of the multiplicity of the mechanisms involved in post-operative pain, a multimodal analgesia regimen with a combination of opioid and non-opioid analgesic drugs is often used to enhance analgesic efficacy and reduce opioid requirements and side-effects.[3]

Gabapentin, a structural analog of gamma aminobutyric acid, is used as an anticonvulsant drug since 1993. In addition, it has been effective in neuropathic pain,[4] diabetic neuropathy,[5] post-herpatic neuralgia,[6] and reflex sympathetic dystrophy.[7] Pre-treatment with gabapentin can block the development of hyperalgesia.[8] Studies have demonstrated that mechanical hyperalgesia surrounding the wound in post-operative patients and experimentally, heat-induced, secondary hyperalgesia share a common mechanism and that central neuronal sensitization contributes to post-operative pain.[9] Gabapentin has a selective effect on the nociceptive process involving central sensitization.[8] Gabapentin and morphine have synergistic analgesic effects in animals and in humans.[10–12] The aim of this study is to know the effect of oral gabapentin given pre-operatively on post-operative pain in patients undergoing abdominal surgeries lasting for around 2 h.

### Aims

To study the effect of oral gabapentin 600 mg given 1 h prior to surgery as preemptive analgesia to attenuate post-operative pain in patients undergoing abdominal surgery under GA.

## MATERIALS AND METHODS

After obtaining the approval of the Institutional Ethics Committee (Grant Medical College and Sir J J Groups of Hospitals) and written informed consent from the patients, 60 patients were included.

### Inclusion criteria

1. Patients belonging to American Society of Anesthesiologists physical status I and II
2. Patients undergoing elective surgery under general anesthesia
3. Anticipated duration of surgery less than 4 h
4. Age group between 18 and 65 years
5. Weight range up to 20% of the ideal body weight for either sex
6. Hemodynamic ally stable

### Exclusion criteria

1. Patients with a history of hypertension, diabetes, and liver disease
2. Patients with acute or chronic renal disease
3. Patients with known neurological disease
4. Neurosurgical and cardiovascular surgical cases
5. Pregnant patients
6. Patients with known psychiatric disorders
7. Patients with anticipated difficult airway
8. Patients on antihypertensive drugs, sedatives, hypnotics, antidepressants, and drugs with effects on the nervous system
9. Patients already taking oral gabapentin
10. Patients allergic to opioids and Tramadol

Patients were randomly divided into two groups, with 30 patients in each group. They were randomly allocated according to computer-generated randomization. Patients in the control group received oral placebo capsules and those in the gabapentin group received 600 mg gabapentin (Neurontin 300 mg capsule; Pfizer, Mumbai, India) 1 h before surgery. The study drugs were prepared by the pharmacy and an appropriate code number was assigned.

In the operating room, a crystalloid infusion was started through an IV cannula. Blood pressure (MAP), heart rate (HR), and peripheral oxygen saturation (SpO_2_) were monitored. For pre-medication, midazolam 0.07 mg/kg, glycopyrolate 0.004 mg/kg, and fentanyl 2µg/kg were administered intravenously on the table. After adequate pre-oxygenation for 3 min, anesthesia was induced with propofol 2 mg/kg and vecuronium 0.1 mg/kg and maintained with 60% N_2_O in O_2_ on Bain’s circuit and vecuronium 0.02 mg/kg. At the end of surgery, neuromuscular block was antagonized with neostigmine 0.05 mg/kg and glycopyrolate 0.008 mg/kg. Surgeries lasting for more than 1 h were given a repeat dose of fentanyl 1µg/kg at the end of the first hour of induction. Any increase in the HR and MAP when not settling down by deepening the plain of anesthesia was given fentanyl 0.05 µg/kg subsequently. Total duration of the surgery in both the groups and the intra-operative fentanyl in both the groups were studied.

After tracheal extubation, patients were observed in the PACU for 1 h and then transferred to the surgical ward. Assessment of post-operative pain was made on the basis of the visual analog score (VAS), where 0 cm - “no pain” and 10 cm - “worst pain imaginable.” The VAS scores are taken immediately on extubation (0 h) and at 2, 4 6, 12, and 24 hours post-operatively.

Post-operative analgesia was provided with Tramadol intravenously. The first dose was given in the PACU as 2 mg/kg and repeated at 8 and 16 h. If the patient still complained of pain, then rescue analgesia was given with diclofenac 75 mg slow intravenous over 10 min. The number of doses of rescue analgesia in both the groups were studied and compared. The occurrence of any side-effects, such as nausea and vomiting, respiratory depression, dizziness, somnolence, headache, and pruritus was recorded. Tramadol dose was omitted if the patient had a respiratory rate <12 breaths/min, an SpO_2_ measured by pulse oximetry <95%, or a serious adverse event related to opioid administration.

## RESULTS

As seen in Tables 1, 2, 2a and 3, patients in both the groups are comparable in terms of demographic parameters like age, sex ratio, and weight.

**Table 1:** Independent *t*-test for equality of mean age in two study groups

Group	N	Mean	SD	*t*	Df	*P*-value	Significance
Gabapentin 600 mg	30	37.8	13.2	−0.805	58	0.424	Not significant
Control	30	40.4	11.8				

**Table 2:** Comparison of sex distribution in two study groups

Group	Treatment	Male (%)	Female (%)	Total
1	Gabapentin 600 mg	12 (40)	18 (60)	30
2	Control	12 (40)	18 (60)	30

**Table 2a:** Chi-square test for comparison of sex in the study groups

Pearson Chi-square	Value	df	*P*-value	Significance
	0.000	1	1.000	Not significant

**Table 3:** Independent *t*-test for equality of mean weight in the two study groups

Group	N	Mean	SD	*t*	Df	*P*-value	Significance
Gabapentin 600 mg	30	55.0	8.1	0.421	58	0.675	Not significant
Control	30	54.2	6.5				

As seen from Tables 4 and 5, there is no significant difference between the groups in terms of total duration of surgery and intraoperative fentanyl usage.

**Table 4:** Independent *t*-test for equality of men duration of surgery in the two groups

Group	N	Mean	SD	*t*	Df	*P*-value	Significance
Gabapentin 600 mg	30	122.0	30.1	0.645	58	0.521	Not significant
Control	30	117.2	27.9				

**Table 5:** Independent *t*-test (mean dose of inj. Fentanyl in µg)

Group	N	Mean	SD	*t*	Df	*P*-value	Significance
Gabapentin 600 mg	30	146.3	32.3	−0.258	58	0.798	Not significant
Control	30	148.3	27.7				

**Figure 1:**
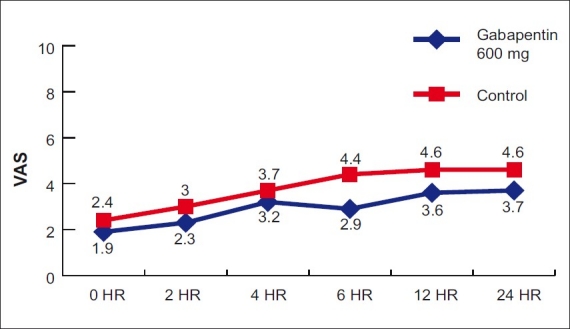
Graph comparing visual analog score in two groups

As seen from Tables 6 and 7 and Figure 1, the VAS scores are significantly lower in the gabapentin 600 mg group as compared to placebo at 0, 2, 4, 6, 12, and 24 h of surgery.

**Table 6:** Independent *t*-test (mean VAS)

Vas	Group	N	Mean	SD	t	Df	*P*-value	Significance
VAS 0 h	Gabapentin 600 mg	30	1.9	0.7	−3.227	58	0.002	Significant
	Control	30	2.4	0.7				
VAS 2 h	Gabapentin 600 mg	30	2.3	0.7	−3.928	58	0.000	Significant
	Control	30	3.0	0.7				
VAS 4 h	Gabapentin 600 mg	30	3.2	0.8	−2.850	58	0.006	Significant
	Control	30	3.7	0.7				
VAS 6 h	Gabapentin 600 mg	30	2.9	0.7	−9.050	58	0.000	Significant
	Control	30	4.4	0.6				
VAS 12 h	Gabapentin 600 mg	30	3.6	0.6	−6.530	58	0.000	Significant
	Control	30	4.6	0.6				
VAS 24 h	Gabapentin 600 mg	30	3.7	0.7	−5.046	58	0.000	Significant
	Control	30	4.6	0.6				

VAS: Visual analog score

**Table 7:** Comparison of the VAS between the study groups (Repeated measure ANOVA)

Vas	Gabapentin 600 mg				Control		
	Mean		SD		Mean		SD
VAS 0 h	1.9		0.7		2.4		0.7
VAS 2 h	2.3		0.7		3.0		0.7
VAS 4 h	3.2		0.8		3.7		0.7
VAS 6 h	2.9		0.7		4.4		0.6
VAS 12 h	3.6		0.6		4.6		0.6
VAS 24 h	3.7		0.7		4.6		0.6

**Group**	**Treatment**	**N**	**Sum of squares**	**df**	**F**	***P*-value**	**Significance**
1	Gabapentin 600 mg	30	66.74	1	113.2	0.000	Significant
2	Control	30					

VAS: Visual analog score

**Figure 2:**
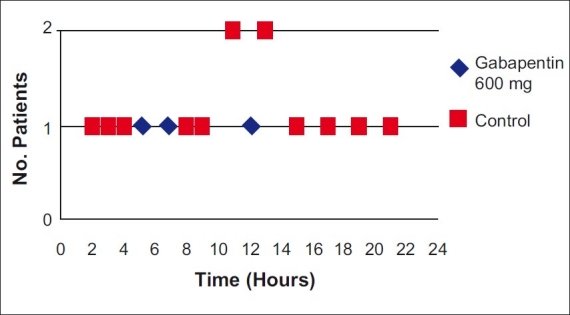
Rescue analgesia requirement in both groups in post-operative period

As seen from Table 8, 8a and Figure 2, three patients in the gabapentin group required rescue analgesia in terms of diclofenac intravenously as compared to 13 patients in the placebo group. The three patients in the gabapentin group requiring rescue analgesia were at around 4, 7, and 12 h after surgery, respectively. Of the 13 patients requiring rescue analgesia, only one patient required it twice, at 4 and 16 h post-surgery, respectively. But, as seen, the rescue analgesia requirement in the gabapentin group was significantly lower as compared to the placebo group.

**Table 8:** Rescue analgesia requirement in two study groups

Group	Treatment	No (%)	Yes (%)	Total
1	Gabapentin 600 mg	27 (90.00)	3 (10.00)	30
2	Control	17 (56.67)	13 (43.33)	30

Figures in parentheses are in percentage

**Table 8a:** Chi-square test for comparison of rescue analgesia in the study groups

Pearson chi-square	Value	df	*P*-value	Significance
	8.523	1	0.004	Significant

## DISCUSSION

The results of our pre-operative oral single-dose study investigating the acute post-operative analgesic effects of gabapentin in patients undergoing abdominal surgeries requiring general anesthesia show that gabapentin decreased post-operative pain scores at 0, 2, 4, 6, 12, and 24 h of surgery and decreased requirement of rescue analgesia when compared with placebo.

Post-operative pain is not purely nociceptive in nature and may consist of inflammatory, neurogenic, and visceral components. Therefore, multimodal analgesic techniques utilizing a number of drugs acting on different analgesic mechanisms are becoming increasingly popular.[1] The main aim in combining different analgesic drugs is to obtain synergistic or additive analgesia, allowing a smaller dose of each drug with an improved safety profile. This can be achieved by combining analgesics acting at different locations (*e.g*., centrally and peripherally acting analgesics). We used Tramadol as a fixed-dose regimen for postoperative pain control and Diclofenac as rescue analgesia to be given only when the patient demanded for it. Tramadol is widely used for moderate-to-severe post-operative pain. Its efficacy is a result of two complementary mechanisms of action: stimulation of µ-opioid receptors and inhibition of norepinephrine and 5-hydroxytryptamine reuptake in pain pathways.[13]

Non-steroidal anti-inflammatory drugs are commonly used analgesics for minor surgery and are useful adjunctive analgesics in patients undergoing major surgery, decreasing pain and opioid requirements. They are well established, effective, and inexpensive. However, their use may be limited by adverse renal, gastrointestinal, and hemostatic effects.

Antihyperalgesic drugs such as gabapentin may have a role in post-operative pain, and the combination with other antinociceptive drugs may produce synergistic analgesia effects.[9,10]

Significantly low VAS scores immediately after extubation, i.e. 0 h (1.9 *vs*. 2.4, *P*=0.002) in the gabapentin group may suggest the synergistic effect of gabapentin with fentanyl. Decreased rescue analgesic requirement in the gabapentin group also suggests the synergistic effect of gabapentin with Tramadol.

Turan *et al*, in 2004, also found that 1,200 mg gabapentin given 1 h prior to surgery significantly reduced the post-operative pain scores and Tramadol requirements in Total abdominal hysterectomy patients.

In animal models of nociception, gabapentin reduces the hypersensitivity associated with nerve injury, inflammation, and pain after surgery.[8,9] Mechanical hyperalgesia surrounding the wound in post-operative patients and experimental, heat-induced secondary hyperalgesia share a common mechanism -central neuronal sensitization, which may contribute to some aspects of post-operative pain. Antihyperalgesic drugs such as gabapentin may have a role in post-operative pain, and the combination with other antinociceptive drugs may produce synergistic analgesia effects.[9,10] Gabapentin enhanced the analgesic effect of morphine in healthy volunteers,[11] and the combination produced better analgesia in comparison with morphine alone in patients suffering neuropathic cancer pain.[12]

Gabapentin significantly decreased morphine consumption and pain in patients after mastectomy[13] although the patients were evaluated for only 4 h after surgery.

Gabapentin is well tolerated. It has few side-effects and minor interactions with other drugs when used for the treatment of chronic pain.[7] We did not observe any significant side-effects associated with a single oral dose of gabapentin. Our results are similar to other published reports.[14–17]

## CONCLUSION

Preemptive use of gabapentin significantly decreases the post-operative pain and rescue analgesic requirement in patients undergoing abdominal surgery under general anesthesia.
